# Microbial Community Restructuring Enhances Composting Efficiency: Synergistic Roles of Thermal Cycling and Fungal Inoculants ( *Fomes lignosus* and *Penicillium glabrum* ) in Metabolic Adaptation

**DOI:** 10.1111/1751-7915.70290

**Published:** 2025-12-26

**Authors:** Yukun Chen, Xiaofang Gong, Xiaobin Xiong, Gangjin Liu, Zhiye Wang, Ying Zhu

**Affiliations:** ^1^ Key Laboratory of Microbial Resources Exploitation and Application of Gansu Province, Institute of Biology, Gansu Academy of Sciences Lanzhou China; ^2^ State Key Laboratory of Herbage Improvement and Grassland Agro‐Ecosystems, College of Ecology, Lanzhou University Lanzhou China; ^3^ Biop Scientific Instruments (Zhejiang) Co., Ltd. Ningbo China

**Keywords:** functional differentiation, humification enhancement, microbial community restructuring, respiratory hierarchy convergence, temperature‐phased aerobic system

## Abstract

This study demonstrates that synergistic integration of thermal cycling (28°C–58°C) and fungal inoculants (*Fomes lignosus*, *Penicillium glabrum*) enhances humification in cattle manure composting by restructuring microbial communities toward metabolic adaptation. Through a temperature‐phased aerobic system, both inoculants significantly improved carbon conversion efficiency, with *F. lignosus* (B) and *P. glabrum* (G) increasing total organic matter by 4.71% and 3.42% (vs. control), humic acid content by 4.58‐fold and 2.35‐fold, and FDA hydrolase activity by 3.28‐fold and 1.22‐fold, respectively, confirming improved humification and nutrient cycling. Temperature–inoculant synergy drove functional differentiation. Respiratory profiling revealed that *P. glabrum* enhanced oxygen consumption by 1.3‐fold during the early thermophilic phase (0–168 h at 58°C). Subsequently, temperature‐induced respiration hierarchies (control > B > G) converged over time with microbial domestication. High‐throughput sequencing and network analyses revealed that temperature–inoculant synergy reshaped the microbiome into simplified consortia, which comprise seven dominant bacterial phyla (e.g., *Firmicutes*, *Actinobacteriota*) and three dominant fungal phyla (e.g., *Ascomycota*), with marked functional differentiation characteristics. *P. glabrum* selectively enriched humification‐related taxa, providing regulatory strategies for enhanced carbon stabilization; whereas *F. lignosus* favoured lignocellulose‐degrading communities, optimising substrate valorisation efficiency. This strategy establishes a targeted microbial framework for optimising the resource utilisation efficiency of lignocellulosic waste within fermentation systems, thereby contributing to circular bioeconomy goals in sustainable organic waste management.

## Graphical abstract

Thermal cycling synergized with fungal inoculants (*Fomes lignosus*/*Penicillium glabrum*) reshapes microbial consortia to boost humification in manure composting, enhancing carbon conversion by 4.71% and humic acid yield 4.58‐fold. This strategy advances lignocellulosic waste valorisation for circular bioeconomy.

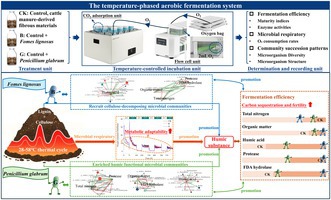

## 1 Introduction

As major livestock‐raising countries, China, India, and Brazil generate substantial cattle manure outputs annually, creating a critical demand for efficient organic waste composting technologies. According to the most recent report from the USDA Global Agricultural Information Network (GAIN, CH2024‐0107), the cattle population in China remained stable at approximately 100 million head between 2023 and January 2025. This vast livestock population generates an enormous biomass output, with cattle manure contributing particularly high daily emissions. Specifically, a single beef cattle can produce 5%–6% of its body weight per day, whereas an adult dairy cow produces an average of 15 kg of manure daily (Wu et al. 2023). The natural degradation rate of cattle manure is slow, and long‐term accumulation poses several environmental hazards, including groundwater contamination, ammonia volatilization, and pathogenic microorganism transmission (Wang et al. 2024; Wu and Bao 2023). Therefore, the efficient and environmentally sound utilisation of cattle manure has become a critical challenge in contemporary agricultural waste management.

**FIGURE 1:**
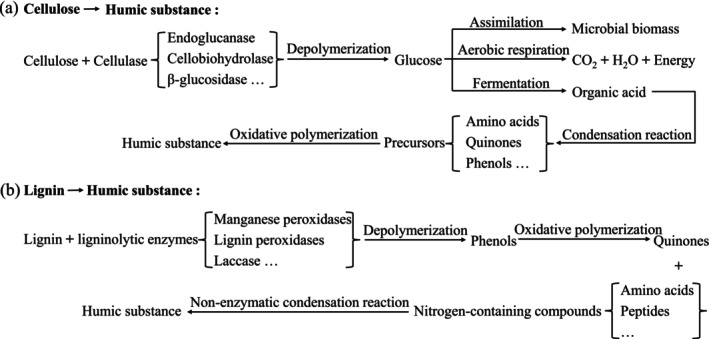
Dominant pathways of lignocellulose humification during composting: (a) Cellulose‐based humification pathways; (b) Lignin‐based humification pathways.

Cattle manure is a typical organic solid waste in agriculture and animal husbandry. Globally, its primary utilisation pathway is fertiliser production. However, the recalcitrant lignocellulosic matrix, which comprises 25%–40% cellulose and 8%–18% lignin (Li et al. 2022), imposes intrinsic constraints on conventional composting systems. These limitations result in prolonged fermentation cycles, low humification efficiency, and substantial nutrient leaching (Yang et al. 2025), collectively leading to the underutilization of approximately 230 million tons of biomass annually. The humification process involves two synergistic pathways (Liu et al. 2024; Jiang, Li, et al. 2021): (a) In the sugar–amine condensation pathway, cellulases depolymerize cellulose into sugars (Fang et al. 2019). Microbial metabolism generates organic acids, acidifying the microenvironment and favouring phenolic/quinonoid precursor accumulation. These undergo non‐enzymatic Maillard reactions with amino compounds to form stable humic substances via oxidative polymerisation (Figure 1a). (b) In the lignin–polyphenol pathway, fungal enzymes, mainly including lignin peroxidase, manganese peroxidases and laccases (Mishra and Jana 2019; Wei et al. 2019), depolymerize lignin into phenolic monomers. These intermediates are oxidised into quinonoid compounds that spontaneously condense with nitrogenous compounds (e.g., amino acids), forming humic acid polymers (Figure 1b). This framework reveals critical interdependencies between biotic (enzymatic depolymerization) and abiotic (chemical polymerisation) phases of humification. However, native microbial consortia often exhibit insufficient ligninolytic capacity, necessitating the strategic design of targeted microbial consortia. Bioaugmentation with selected ligninolytic fungi and cellulolytic bacteria has emerged as a promising strategy to enhance humic acid formation and shorten composting durations, thereby aligning with objectives in both resource recovery and environmental sustainability.

Recent advances in microbial inoculant technologies have demonstrated significant promise for the efficient composting of organic waste (Mitra et al. 2024). Cutting‐edge research shows that specific microbial strains can markedly accelerate organic matter decomposition and promote humification. For example, Yang et al. (2023) reported that a composite inoculant containing *Bacillus* spp., *Yeast in the ellipse* Yj‐5, and *Aspergillus oryzae* MQ1 significantly improved thermophilic phase initiation and organic matter degradation in cattle manure composting. Similarly, Wang et al. (2011) demonstrated that the lignocellulolytic fungus *Penicillium expansum* W4 enhanced lignocellulose degradation by 57.5% and increased humus content by 12.0%. Brown‐rot fungi have also been shown to accelerate compost maturation and enhance the activity of lignocellulolytic enzymes, thereby improving the nutrient quality of compost products (Li, Liu, et al. 2025; Zhu et al. 2021). These studies provide a robust theoretical foundation for optimizing microbial‐augmented composting technologies.

White‐rot fungi and *Penicillium* species are two phylogenetically distinct fungal groups with exceptional lignocellulose‐degrading capacities. White‐rot fungi, known as “natural engineers” of lignin degradation, selectively cleave lignin and remove its barrier over cellulose by secreting lignin peroxidase (LiP), manganese peroxidase (MnP), and laccase (Lac) (Pradeep Kumar et al. 2023). In contrast, *Penicillium* spp. are characterised by high cellulase and hemicellulase activities; their β‐glucosidase activity effectively alleviates inhibition of enzyme activities by cellobiose (Xie et al. 2023). Through strain screening, two high‐performance isolates were identified: the white‐rot fungus *Fomes lignosus* (GSICC 60654) and *Penicillium glabrum* (GSICC 61611). Under solid‐state fermentation, *F. lignosus* exhibited a 4.5‐fold increase in lignin degradation (Klason lignin assay), whereas *P. glabrum* achieved 2.3‐fold higher holocellulose degradation than uninoculated controls. Notably, their co‐culture system shows significant synergistic effects. However, the functional mechanisms and composting benefits of these strains in real‐world systems remain inadequately characterised, limiting their transition from laboratory‐scale validation to industrial‐scale implementation.

Hence, the present study employed a low‐temperature–high‐temperature cyclic acclimation aerobic fermentation system to systematically evaluate the effects of inoculating *F. lignosus* or *P. glabrum* on fermentation performance, microbial metabolic activity, and community dynamics in cattle manure‐derived fibrous materials under controlled laboratory conditions. The following hypotheses were proposed: (1) fungal inoculation with *F. lignosus* or *P. glabrum* will significantly enhance the fermentation efficiency of cattle manure‐derived fibrous materials; (2) these inoculants will stimulate microbial respiratory metabolism to accelerate lignocellulose bioconversion; (3) the introduced fungi will functionally restructure the compost microbiome, modulating both microbial diversity and keystone taxa; and (4) *F. lignosus* and *P. glabrum* will exhibit divergent yet complementary roles in optimizing fermentation outcomes, particularly in humification. This study contributes to the current knowledge base by (i) quantitatively assessing the scalability of fungal inoculants for industrial composting of cattle manure, (ii) elucidating mechanistic interactions between exogenous fungi and indigenous microbial communities, and (iii) proposing a novel temperature‐cycling acclimation strategy to enhance fungal adaptation under non‐isothermal composting conditions. The results are expected to inform the rational design of bioaugmentation protocols, advancing the sustainable conversion of agricultural waste into high‐value biofertilizers and supporting the development of circular agro‐industrial systems within the resource–product–renewable resource framework.

## 2 Materials and Methods

### 2.1 Preparation of Cattle Manure‐Derived Fibrous Materials

Fresh cattle manure was aseptically collected from the intensive beef cattle facility operated by Gansu Huarui Agriculture Co. Ltd. To optimize the carbon‐to‐nitrogen (C/N) ratio for subsequent processing, crushed corn stover (0.5–1 cm) was immediately incorporated into the manure, adjusting the mixture to a target C/N of 25:1. This composite material was then subjected to natural shade‐drying for 72 h under ambient conditions (25°C ± 2°C; relative humidity 30%–50%). Post‐drying analysis confirmed a final moisture content of 2%. The resulting cattle manure‐derived fibrous materials were homogenized to ensure physicochemical consistency, vacuum‐sealed in sterile barrier bags, and stored in desiccators containing silica gel (SiO^2^) at 25°C until further experimental use.

### 2.2 Fungal Strains and Inoculum Development

The experimental fungal strains included *F. lignosus* (preservation number GSICC 60654) and *P. glabrum* (preservation number GSICC 61611), which were selected based on preliminary screening for their cellulolytic activity and biocontrol potential. All strains were preserved at the Gansu Branch of the China Industrial Microorganism Collection and Management Center (GSICC, accessible via http://jzk.gsmsc.cn/).

Inoculum preparation began with sterilisation of the substrate, which consists of commercial rice mixed with deionised water at a 1:1 (w/v) ratio. The mixture was distributed in 200 g aliquots into 500 mL wide‐mouth bottles equipped with breathable lids and sterilised by autoclaving (121°C, 20 min). After the system was cooled to 25.0°C ± 0.5°C, each bottle was aseptically inoculated with a spore suspension of either *F. lignosus* or *P. glabrum* . The inoculation concentration of viable spores was determined via the gradient dilution plate spreading method. Specifically, the number of viable spores was quantified as Colony‐Forming Unit (CFU), and the final inoculation concentration was controlled at 1 × 10^6^ viable spores/g substrate. Solid‐state fermentation proceeded under dark, static conditions for 7 days at 25°C ± 0.5°C. After incubation, the developed mycelial biomass was aseptically harvested, transferred to pre‐sterilised 10 mL centrifuge tubes, and stored at 4°C for subsequent use as bioinoculants.

### 2.3 Incubation Design and Material Collection

The Gas Endeavour breathing instrument system (BPC Instruments AB, Lund, Sweden) was used to conduct a low‐temperature–high‐temperature cyclic acclimation aerobic fermentation experiment. This experiment aimed to investigate the effects of *F. lignosus* and *P. glabrum* inoculation on microbial respiratory metabolism during the degradation of cattle manure‐derived fibrous materials. Three treatment groups were established: CK (control, 50 g of dried cattle manure‐derived fibrous material plus 100 mL of sterile distilled water), B (CK material fortified with 9.5 g of *F. lignosus*), and G (CK material fortified with 9.5 g of *P. glabrum* ). Each treatment included three replicates. All materials were placed into 500 mL Duran flasks and incubated under a three‐stage cyclic temperature regime: 28°C for 72 h → 58°C for 96 h → 28°C for 72 h. One full cycle lasted 7 days and was repeated four times, for a total incubation period of 28 days. This cyclic regime promoted fungal colonisation and activation at low temperature (28°C), simulated industrial composting conditions for enhanced lignocellulose degradation at high temperature (58°C), and facilitated humus stabilisation during the final cooling phase. This design aimed to ensure uniform maturation, optimise microbial colonisation, and enhance the overall transformation of substrates into value‐added products.

Oxygen consumption was continuously monitored using the Gas Endeavour system, which measured real‐time gas production via water displacement (Pagliaccia et al. 2019). For each 2 mL of gas generated, the system automatically released and recorded the gas volume, with environmental parameters (temperature and pressure) synchronised and standardised to reference conditions (0°C, 1 atm, and zero moisture content). Gas volumes were recorded as normalised millilitres (NmL), and data were collected hourly. At the end of the incubation period, 10 g of fermented material was aseptically collected and stored at −80°C for microbiome sequencing analysis (16S rRNA and ITS). The remaining material was divided for various analyses and stored at either 4°C (total nitrogen, organic matter, humic acid) or −20°C (protease and FDA hydrolase activity).

### 2.4 Determination of Nutrients and Enzymes Parameters

The physicochemical and enzymatic properties of all samples were systematically analysed. Total nitrogen was quantified using a modified Kjeldahl nitrogen determination method (0.5 g sample, digestion at 420°C for 2 h). Organic matter content was determined using the Walkley–Black method (0.2 g sample, oil bath at 170°C for 30 min). Humic acid was sequentially extracted using sodium pyrophosphate and sodium hydroxide (5.0 g sample, water bath at 65°C for 1 h), and content was determined using potassium dichromate oxidation. Enzyme activities were measured as follows: (1) Protease activity was assayed using casein as a substrate in Tris–HCl buffer (50°C, 2 h), followed by spectrophotometric detection at 570 nm; results were expressed in U·g^−1^. (2) FDA hydrolase activity was assessed via dark oscillation at 37°C for 3 h, followed by fluorescence detection (excitation/emission at 490/515 nm); results were reported in μg·g^−1^·h^−1^. All measurements were performed in triplicate using instruments such as FOSS Kjeltec 8400 and the Varioskan LUX microplate reader.

### 2.5 Genomic DNA Extraction and Sequencing Analysis

High‐throughput sequencing of bacterial and fungal communities was conducted on samples from each treatment. Genomic DNA was extracted using the MoBio PowerSoil DNA Isolation Kit (12888). For bacterial community analysis, the V3–V4 region of the 16S rRNA gene was amplified using primers 338F (ACTCCTACGGGGAGGCAGCAG) and 806R (GGACTACHVGGGTWTCTAAT). For fungal community analysis, ITS1F (5′‐CTTGGTTCATTTAGGAGAGTAAGTAA‐3′) and ITS2R (5′‐GCTGTGTTCATCGATGC‐3′) primers were used. Operational taxonomic units (OTUs) were clustered based on 97% sequence similarity. Bioinformatics analysis was performed using the online platform of Shanghai Majorbio Bio‐Pharm Technology Co. Ltd. All analyses were conducted in triplicate.

### 2.6 Statistical Analysis

One‐way analysis of variance (ANOVA) was performed to assess statistical significance using SPSS 20.0 software (SPSS Inc., Chicago, USA). Graphs of physicochemical parameters were generated using OriginPro 9.1. Redundancy analysis (RDA) and microbial network analysis were carried out using the online platform of Shanghai Majorbio Bio‐Pharm Technology Co. Ltd.

## 3 Results and Discussion

### 3.1 Nutrients and Enzymes Properties

Our systematic evaluation of the temperature‐acclimated aerobic fermentation system revealed distinct trends in nutrient transformation and enzymatic activity (Table 1). Total nitrogen (TN) content declined significantly in the bioaugmented groups (CK: 0.47% ± 0.017% > G: 0.44% ± 0.010% > B: 0.39% ± 0.044%), with treatment B showing a 17.02% reduction. The degradation of cellulose and hemicellulose provided essential energy and carbon sources for the activity of nitrogen‐transforming microorganisms (Liu et al. 2025). The significant decrease in total nitrogen content of the material during this process may have been attributed to enhanced microbial assimilation of nitrogen induced by fungal inoculation (Hoang et al. 2022; Li, Liu, et al. 2025), with *F. lignosus* demonstrating particularly effective performance. The synergistic accumulation of organic matter (OM) and humic acid (HA) indicated that fungal augmentation significantly promoted humification (B > G > CK). HA content reached 5.82% ± 0.068% in treatment B (4.58‐fold that of CK) and 2.98% ± 0.226% in treatment G (2.35‐fold that of CK), mirroring the OM accumulation pattern. A strong correlation was observed between OM and HA content (*R* ^2^ = 0.917, *p* < 0.01), reflecting advanced humification processes, demonstrating the enzymatic cascade model in which fungal‐derived enzymes catalyse phenolic polymerisation, thereby accelerating the mechanism of OM‐to‐HA conversion consistent with the humification pathway reported by Li, Li, and Sun (2025). These outcomes underscore the role of fungal inoculants in accelerating organic matter maturation through microbial community restructuring and metabolic stimulation.

**TABLE 1:** Nutrients and enzymes properties of aerobic fermentation.

Treatments	Total nitrogen content (%)	Organic matter content (%)	Humic acid content (%)	Protease activity (U·g^−1^)	FDA hydrolase activity (μg·g^−1^·h^−1^)
CK	0.47 ± 0.017a	52.41 ± 0.286b	1.27 ± 0.061c	174.64 ± 8.315a	21.85 ± 1.507c
B	0.39 ± 0.044c	54.88 ± 0.685a	5.82 ± 0.068a	174.64 ± 8.315a	71.76 ± 3.661a
G	0.44 ± 0.010ab	54.20 ± 0.762a	2.98 ± 0.227b	108.11 ± 8.315b	26.73 ± 1.386b

*Note:* Letters (a, b or c) indicate significant differences between treatments at the 5% level based on Duncan's multiple‐range test. Values are presented as means ±standard deviation (*n* = 3).

Protease activity plays a pivotal role in nitrogen cycling by catalysing the hydrolysis of short‐chain polypeptides into ammonia (Ros et al. 2006) and also mediates the breakdown of complex nitrogenous compounds into bioavailable forms (Chen et al. 2003). In this study, protease activity followed a CK = B > G pattern, with suppression observed in treatment G, likely due to shifts in community composition. FDA hydrolase activity, an indicator of total microbial metabolic capacity with known links to carbon and nutrient cycling (Li et al. 2018), was significantly enhanced in bioaugmented groups, increasing by 3.28‐fold in B and 1.22‐fold in G compared to that in CK. This increase aligns with previous findings that elevated TN levels stimulate microbial proliferation and enzymatic activity (Nayak et al. 2007), reinforcing the conclusion that fungal bioaugmentation enhances microbial functional potential within composting environments.

### 3.2 Respiratory Activity of Microorganisms

**FIGURE 2:**
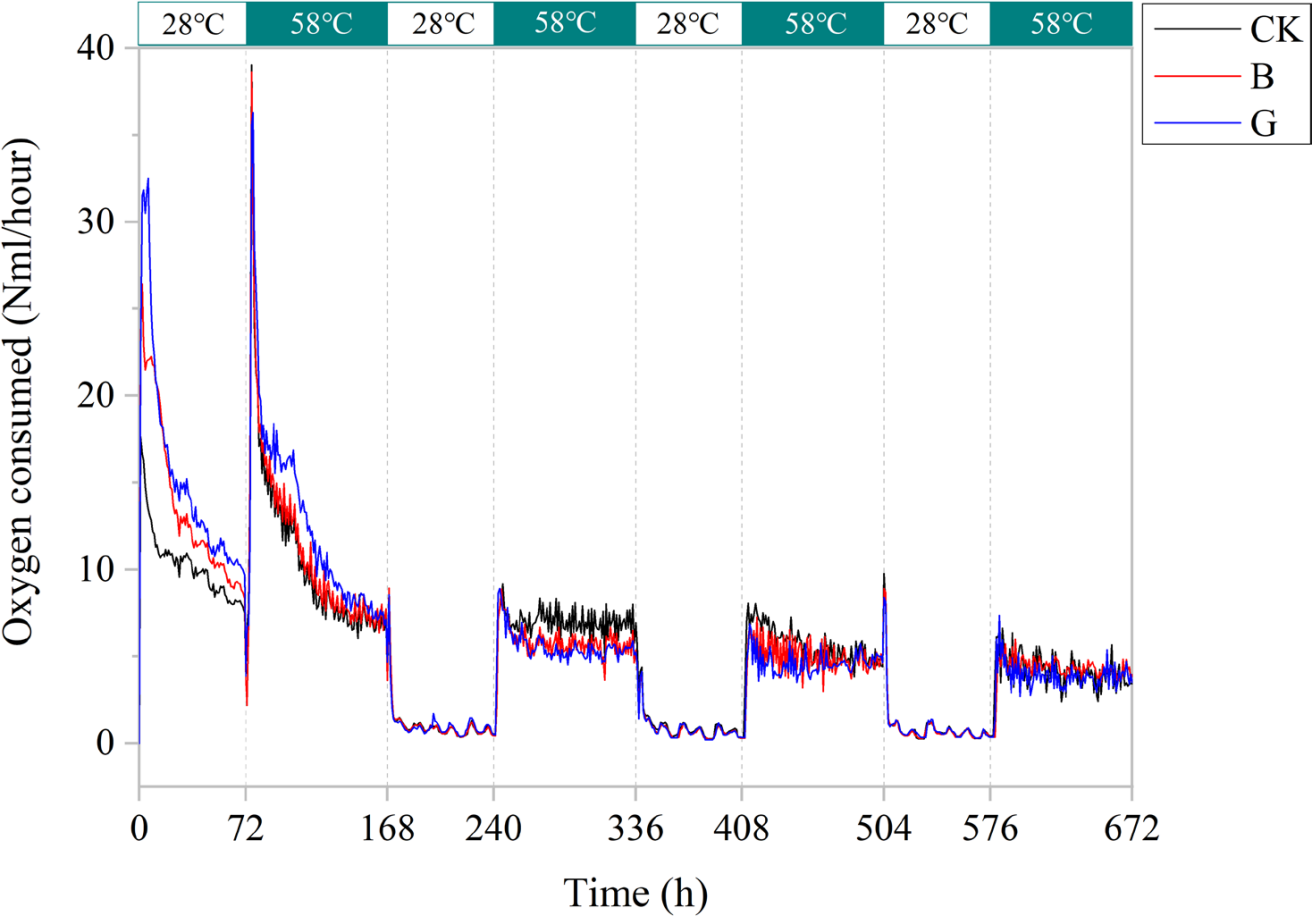
Dynamic changes in oxygen consumption in aerobic fermentation system under temperature cycle conditions.

Real‐time monitoring of oxygen uptake under alternating thermal regimes (28°C–58°C) revealed dual‐phase impacts of fungal inoculants (*F. lignosus* [B] and *P. glabrum* [G]) on microbial respiratory metabolism during degradation of cattle manure‐derived fibrous materials (Figure 2). Respiratory dynamics reveal the temporal characteristics of metabolism (Schmitz et al. 2024). During the early thermophilic phase (0–168 h at 58°C), inoculated treatments showed elevated metabolic activity in the order G > B > CK. Notably, treatment G exhibited a 1.3‐fold increase in oxygen consumption compared to CK (*p* < 0.05). It demonstrated the advantages of G treatment in the early stage of high‐temperature fermentation, which is conducive to activating the humification chain reaction and enhancing the mineralization of organic matter (Greff et al. 2022). These results demonstrate that temperature cycling operates via two synergistic mechanisms: (1) fungal inoculants selectively enrich thermotolerant taxa such as *Thermus* spp., consistent with the thermotolerant taxa selection mechanism reported by Zhu et al. (2021) for fungal at the thermophilic phase; and (2) cyclical transitions between 28°C and 58°C drive consortium adaptation and stabilization, as raising the composting temperature supports the reproduction of thermophilic bacteria that release heat during biomass decomposition, creating a positive feedback effect which accelerates organic matter decomposition (Adamski et al. 2024). However, due to the fact that lignin degradation depends on mesophilic enzyme activity (Dutta et al. 2025), the peak of humic acid accumulation lags behind the respiratory peak of treatment G, resulting in treatment B (*F. lignosus* inoculation) showing significant efficacy at the humification maturity stage. Temperature‐stratified analysis further revealed that the 58°C phase induced microbial community restructuring (CK > B > G), whereas the 28°C phase fostered equilibrium in metabolic function. As the process progressed, functional convergence became apparent, with G, B, and CK showing increasingly similar respiration profiles in later phases. Thus, temperature oscillation promoted short‐term functional enhancement via fungal activation and long‐term compositional resilience via microbial succession.

### 3.3 Diversity of Bacterial and Fungal Communities

**FIGURE 3:**
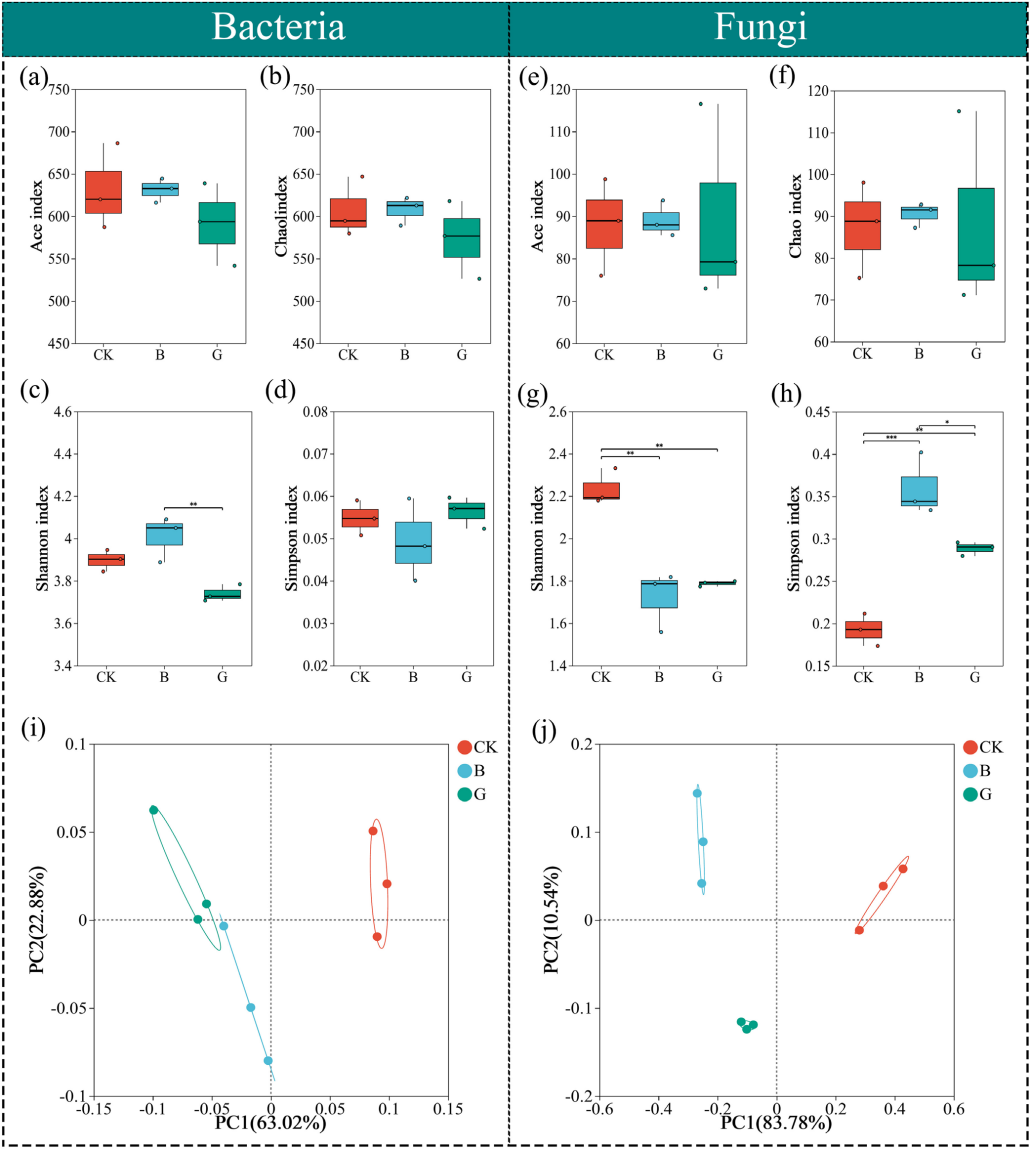
Alpha and beta diversity of microbial communities. (a–h) Alpha diversity indices for bacteria: Ace (a), Chao1 (b), Shannon (c), and Simpson (d), and for fungi: Ace (e), Chao1 (f), Shannon (g), and Simpson (h). Different letters indicate significant differences (*p* < 0.05). (i–j) Beta diversity based on Weighted UniFrac for bacterial (i) and fungal (j) communities.

Microbial community richness and diversity were evaluated at the OTU level using α‐diversity indices (Chao1, Ace, Shannon, and Simpson) (Figure 3). In the bacterial community, treatment B significantly enhanced both richness (Chao1 and Ace: B > CK > G) and diversity (Shannon: B > CK > G, *p* < 0.05), whereas the Simpson index showed the inverse trend (B < CK < G), confirming the promotion of microbial complexity by *F. lignosus* inoculation. In contrast, fungal communities exhibited more complex responses. Richness indices presented varied trends (Chao1: B > CK > G; Ace: CK > B > G), whereas diversity metrics consistently decreased in bioaugmented treatments (Shannon: CK > B ≈ G; Simpson: B > G > CK, *p* < 0.05), indicating a community shift that favours functional rather than taxonomic diversity. Beta diversity analysis based on Weighted UniFrac distance revealed high intra‐treatment similarity and clear inter‐treatment separation (ANOSIM: *R* = 0.9424 for bacteria; *R* = 1.0000 for fungi; both *p* = 0.0010), indicating that both fungal inoculants substantially restructured microbial communities. These findings align with previous studies (Dong et al. 2024; Karadag et al. 2013; Matheri et al. 2023), which demonstrate that thermophilic composting enhances microbial diversity and functional specialisation, particularly through enrichment of lignocellulose‐degrading taxa.

### 3.4 Composition of Bacterial and Fungal Communities

High‐throughput sequencing of temperature‐acclimated cattle manure‐derived fibrous materials revealed pronounced microbial compositional shifts in response to fungal inoculants B (*F. lignosus*) and G ( *P. glabrum* ) compared to the uninoculated control (CK). A total of 639,859 quality‐filtered bacterial and 895,819 fungal sequences were obtained. At a 97% similarity threshold, these sequences clustered into 1176 bacterial OTUs (CK: 553; G: 528; B: 566) and 256 fungal OTUs (CK: 84; G: 86; B: 86). The asymptotic rarefaction curves (Figure S1) and universal coverage indices (> 0.99) for all samples confirmed adequate sequencing depth to capture microbial diversity, with bacterial communities particularly well represented.

**FIGURE 4:**
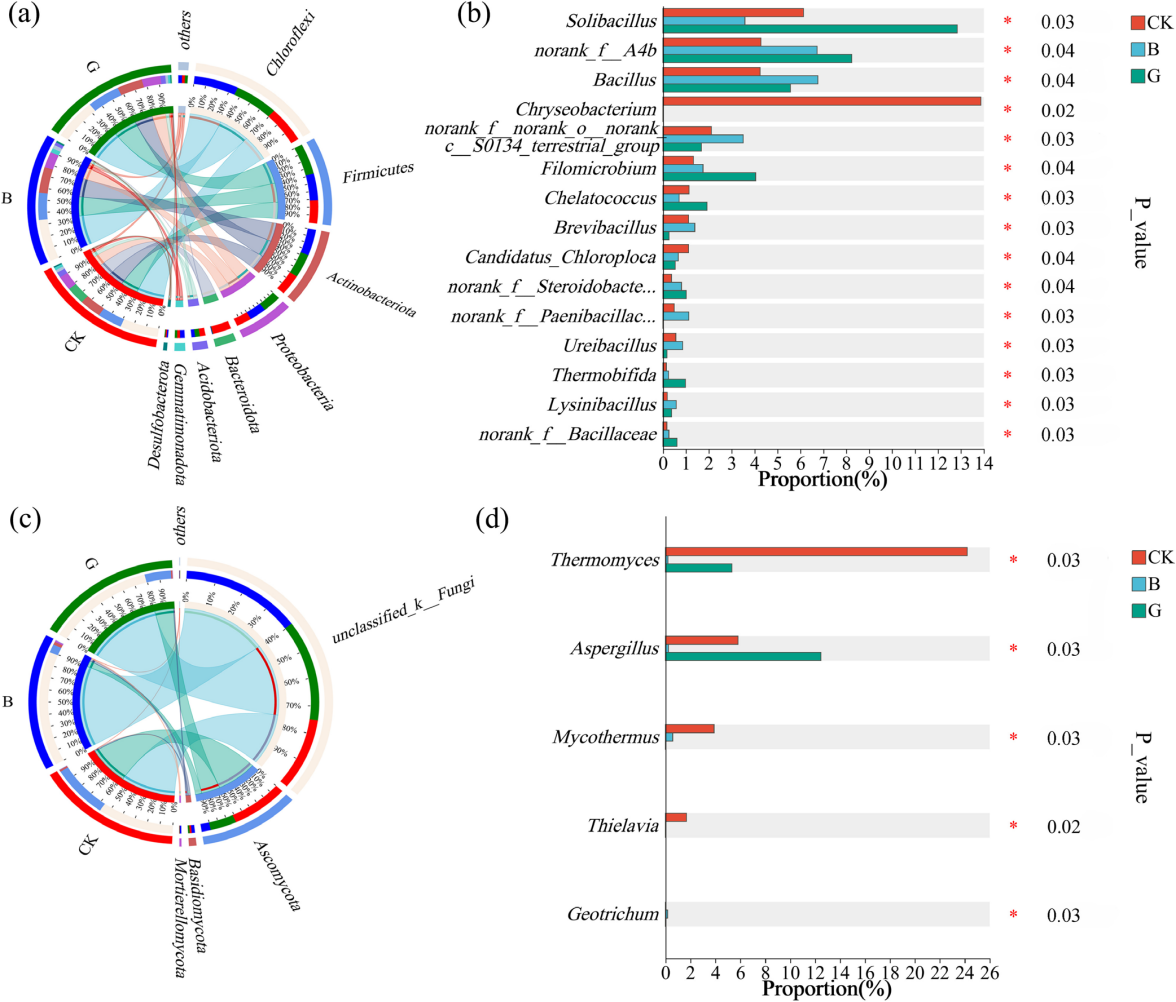
Microbial community composition and differential abundance analysis. Relative abundances at the phylum level for bacteria (a) and fungi (c); Kruskal–Wallis H test bar plots at the genus level for bacteria (b) and fungi (d). Symbols denote significant inter‐group differences (*p* ≤ 0.05); values indicate relative abundance differences among treatments.

Bioaugmentation with inoculants B (*F. lignosus*) and G ( *P. glabrum* ) induced significant restructuring of the thermophilic fermentation microbiota. Phylum‐level bacterial analysis identified eight dominant taxa (> 1% relative abundance, Figure 4a), with thermotolerant phyla—*Chloroflexi* (B: 35%; G: 34%; CK: 30%), *Firmicutes* (B: 33%; G: 38%; CK: 29%), *Actinobacteriota* (B: 37%; G: 34%; CK: 29%), and *Proteobacteria* (B: 33%; G: 37%; CK: 29%)—comprising the majority of the microbial consortia (76.08%–91.73%). These groups are well‐documented for their roles in the degradation of recalcitrant organic compounds and humification during composting (Wei et al. 2018; Jiang et al. 2020; Jiang, Wang, et al. 2021; Xu et al. 2020; Wang et al. 2022). In particular, *Proteobacteria* are notable for thermal adaptability and metabolic gene repertoires supporting amino acid transport, nitrogen fixation, and small‐molecule organic acid metabolism (Xu et al. 2022). Conversely, *Bacteroidota* were nearly eliminated in both B and G (decreased by 98% and 96%, respectively), and *Acidobacteriota* displayed differential susceptibility (reduced by 41% in B, 14% in G). Notably, *Gemmatimonadota* surged by 65% in B but declined by 20% in G, whereas *Desulfobacteriota* increased by 18% in B and declined by 48% in G. These taxa‐specific divergences underscore distinct modes of community modulation by the two inoculants. Treatment B produced more pronounced enrichment of thermophilic, lignocellulose‐degrading taxa, consistent with its greater enhancement of organic matter decomposition and humification processes.

Genus‐level analysis via Kruskal–Wallis *H* test (*p* ≤ 0.05) further clarified the functional differentiation among treatments (Figure 4b), with bioaugmentation agents B and G exhibiting distinct functional recruitment patterns. Treatment B selectively enriched *Brevibacillus*, *norank_f_Paenibacillaceae*, and *Ureibacillus* (*Firmicutes*), which contribute to cellulose degradation via benzoate metabolic pathways (Niu et al. 2021; Ren et al. 2023) and nutrient cycling (Chen et al. 2024; Xiong et al. 2023). In contrast, treatment G promoted *Solibacillus* (*Firmicutes*), associated with humification precursor synthesis (Huang et al. 2019), and *Chelatococcus* (*Proteobacteria*), which mediates carbon‐nitrogen transformation and humus synthesis (Li et al. 2023). Both bioaugmentation treatments significantly suppressed *Chryseobacterium*, likely due to post‐fermentation pH exceeding its optimal growth threshold of 5.0 (He et al. 2019), However, seven functionally important genera were upregulated: *Bacillus*, *norank_f_Bacillaceae*, and *Lysinibacillus* (*Firmicutes*) mediated starch hydrolysis via amylase secretion (Jiang, Wang, et al. 2021), urease‐dependent nitrogen transformation (Yin et al. 2021), and lignocellulose degradation (Duan et al. 2022; Dong et al. 2024; Zhao et al. 2024), respectively. Additionally, *norank_f_A4b* (*Chloroflexi*) facilitated hydrolytic acidification of complex substrates (Jiang et al. 2023; Yuan et al. 2024), whereas *Proteobacteria* members *Filomicrobium* and *norank_f_Steroidobacteraceae* mediated plant cellulose decomposition (Tom et al. 2022) and nitrogen cycle modulation (Shi et al. 2023). *Thermobifida* (*Actinobacteriota*) also increased under inoculation, producing hydrolases that target plant cell wall components to enhance organic mineralization (Gomez del Pulgar and Saadeddin 2014; Xiong et al. 2023). Treatment B showed stronger activation of *Firmicutes*‐linked enzymatic degradation pathways, whereas Treatment G more effectively stimulated humification and nutrient cycling.

Fungal community composition was dominated by four phyla: *unclassified_k_Fungi*, *Ascomycota*, *Basidiomycota*, and *Mortierellomycota* (Figure 4c). Treatments B and G both increased the abundance of *unclassified_k_Fungi* (B: 25% to 40%; G: 25% to 35%) while markedly reducing *Ascomycota* (B: 62% to 10%; G: 62% to 29%). Within *Ascomycota*, both treatments suppressed *Thermomyces*, a major hemicellulase and cellulase producer (Zhang et al. 2015). However, only treatment G significantly enriched *Aspergillus* (Figure 4d), a thermotolerant lignin‐degrading genus linked to humification precursor synthesis (Huang et al. 2019). Treatment B uniquely stimulated *Basidiomycota* (78% increase) and *Mortierellomycota* (7‐fold increase), both recognized for saprophytic lignin degradation (Medina et al. 2020). In contrast, G suppressed *Basidiomycota* (−21%) and eliminated *Mortierellomycota*, suggesting differential competitive dynamics. These patterns indicate that treatment G enhances humification via *Aspergillus*‐mediated lignin transformation, whereas B facilitates lignocellulose decomposition through *Basidiomycota* and *Mortierellomycota* enrichment. The reduced presence of *Ascomycota* in B may result from competitive exclusion by faster‐growing saprophytic fungi.

Overall, the observed microbial shifts at both phylum and genus levels support the conclusion that temperature‐acclimated fungal inoculation restructures microbial networks to enhance fermentation performance. Specifically, *F. lignosus* (B) promoted *Firmicutes*‐dominated lignocellulose degradation, whereas *P. glabrum* (G) favored humification and carbon‐nitrogen cycling, offering complementary strategies to improve fibrous waste bioconversion.

### 3.5 Relationships Between Physicochemical Characteristics, Enzyme Activity and Microorganisms During Composting

**FIGURE 5:**
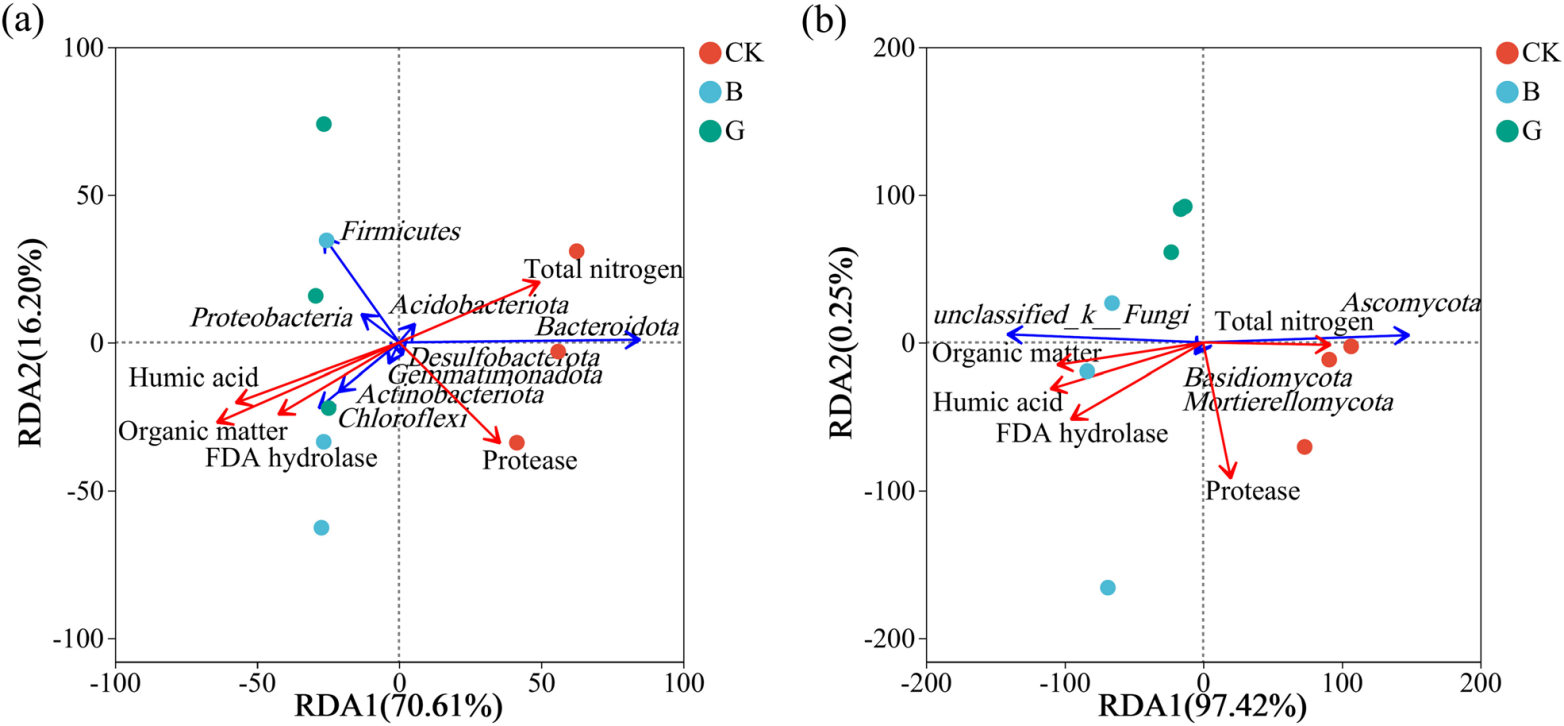
Redundancy analysis (RDA) biplots illustrating bacterial (a) and fungal (b) community relationships with physicochemical and enzymatic variables.

RDA revealed that five key variables—FDA hydrolase activity, organic matter (OM), total nitrogen (TN), protease, and humic acid (HA)—explained 86.81% (70.61% + 16.20%) of bacterial and 97.67% (97.42% + 0.25%) of fungal community variation at the phylum level (Figure 5a,b). For bacteria, OM emerged as the primary driver (*p* < 0.01), indicating a dual role as both a decomposition product and metabolic substrate, followed by HA > TN > FDA > protease. Notably, dominant bacterial phyla such as *Chloroflexi*, *Firmicutes*, *Actinobacteriota*, *Proteobacteria*, *Gemmatimonadota*, and *Desulfobacteriota* showed strong positive correlations with OM, HA, and FDA, consistent with their enrichment in treatments B and G (Figures 3i and 4a). Fungal community shifts were shaped primarily by HA (*p* < 0.01), with OM > FDA > TN > protease exerting secondary influence. *Basidiomycota* and *Mortierellomycota*, ligninolytic phyla enriched under inoculation, showed positive associations with OM, HA, and FDA (Figures 3j and 4c). These findings indicate that microbial assembly was environmentally filtered, with bacterial networks structured primarily by OM‐mediated metabolism and fungal networks shaped via HA‐driven humification.

**FIGURE 6:**
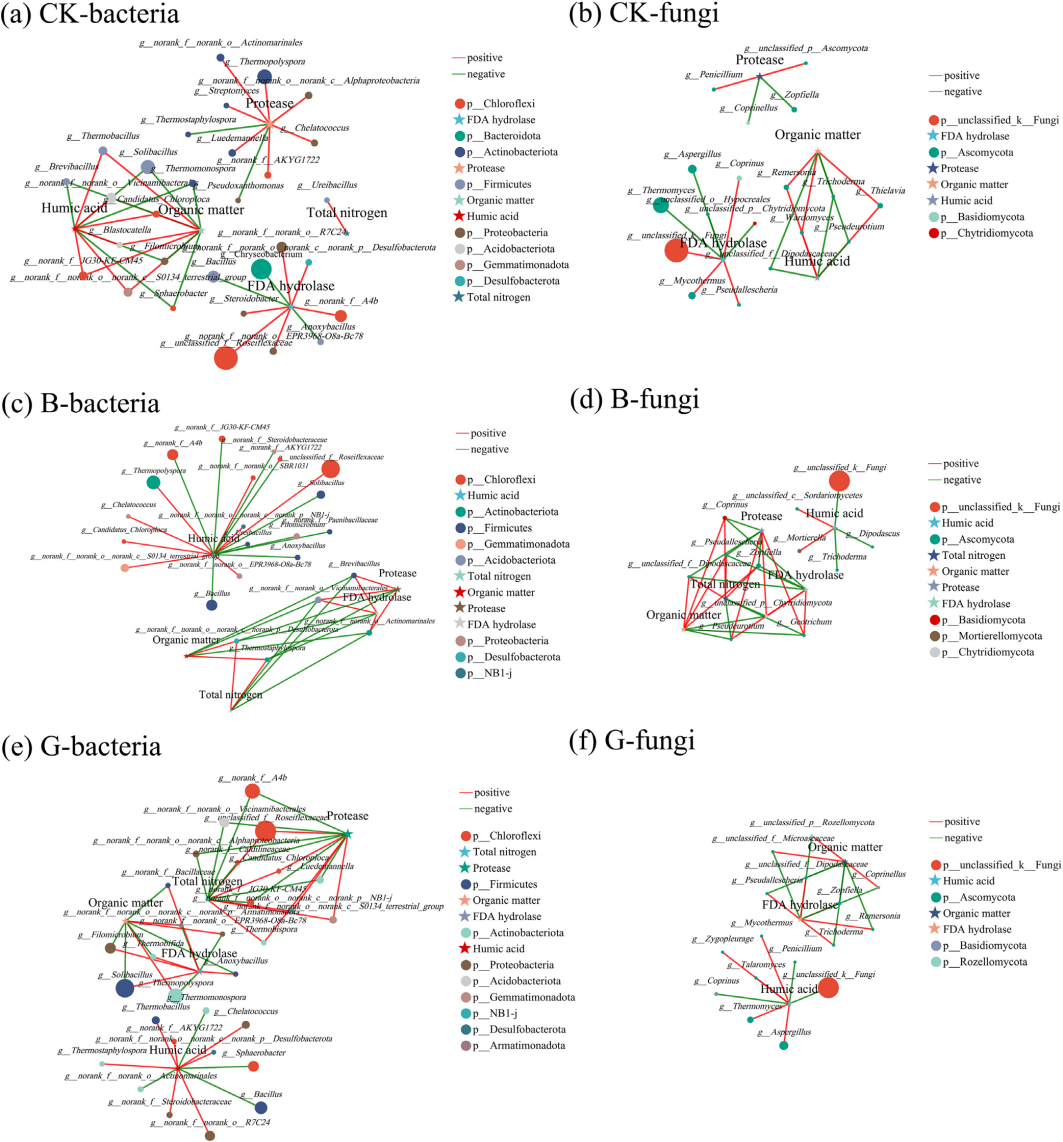
Co‐occurrence network interactions of bacteria (a, c, e) and fungi (b, d, f). Nodes represent operational taxonomic units (OTUs); connections indicate strong (|*r*| > 0.6) and significant (*p* < 0.05) positive correlations. Node colour indicates phylum, size reflects degree (number of connections), and edge thickness represents correlation strength (red: Positive, green: Negative).

Co‐occurrence network analysis (Spearman's |*r*| > 0.6, *p* < 0.05) revealed contrasting structures between bacterial (26 genera) and fungal (30 genera) communities across treatments (Figure 6). Bacterial networks exhibited higher complexity than fungal counterparts, with CK (35 nodes, 41 edges, and 8 phyla), B (28 nodes, 38 edges, and 8 phyla), and G (35 nodes, 49 edges, and 9 phyla), all showing robust connectivity with OM, TN, HA, protease, and FDA. In contrast, fungal networks showed reduced connectivity (CK: 22 nodes, 24 edges, and 4 phyla; B: 17 nodes, 33 edges, and 5 phyla; G: 19 nodes, 24 edges, and 4 phyla) and selective parameter associations (e.g., TN exclusion in CK). Dominant phyla included *Chloroflexi*, *Firmicutes*, *Actinobacteriota*, *Proteobacteria*, *Gemmatimonadota*, *Desulfobacteriota*, and *Acidobacteriota* among bacteria and *Ascomycota*, *Basidiomycota*, and *Mortierellomycota* among fungi. Distinct ecological roles emerged: *Chryseobacterium* (*Bacteroidota*) negatively correlated with FDA in CK; an unclassified *Armatimonadota* genus correlated with OM accumulation in G; and an unclassified *NB1‐j* genus showed positive associations with TN and protease in B and G.

Bioaugmentation induced functional reorganisation of bacterial modules. CK showed four discrete clusters (FDA, protease, OM–HA, and TN), which shifted to three modules (HA, OM–FDA, TN–protease) under G, and collapsed into two hubs (HA, OM–FDA–TN–protease) under B. HA‐positive correlations increased from 54% in CK to 63% in G. These shifts indicate inoculant‐mediated niche partitioning: treatment G promoted humic acid synthesis (63% HA‐linked bacteria) and *Aspergillus*‐mediated humification, whereas treatment B optimised humic acid synthesis (44% HA‐linked bacteria) via *Firmicutes*‐mediated OM degradation and nitrogen cycling (TN‐associated clusters) fungi. The results corroborate thermal adaptation principles: fungal networks undergo simplification under thermophilic stress, whereas bacterial networks exhibit structural resilience and functional integration in lignocellulose degradation.

## 4 Conclusions

This study demonstrates that *F. lignosus* (B) and *P. glabrum* (G) differentially optimise aerobic fermentation of cattle manure‐derived fibrous materials via temperature‐phased (28°C–58°C) metabolic specialisation. *F. lignosus* enhances lignocellulose degradation by increasing cellulolytic *Firmicutes* and *Actinobacteriota* by 27.59% and elevating FDA activity 3.28‐fold, whereas *P. glabrum* promotes humification by enriching Proteobacteria and *Aspergillus*‐mediated humic acid yield by 1.35‐fold. These effects restructure bacterial‐dominant networks into distinct hubs: G forms three (HA, OM‐FDA, TN‐protease), and B consolidates into two (HA, OM‐FDA‐TN‐protease), accelerating carbon stabilisation. This establishes a scalable microbiome engineering framework for circular agriculture, demonstrable at farm scale. Future research should prioritise molecular marker‐assisted thermal adaptation in fungi through targeted engineering of genes encoding thermostable ligninolytic enzymes, particularly laccase (*lcc* genes) and manganese peroxidase (*mnp* genes), to enhance systemic resilience under climate change scenarios.

## Author Contributions

**Yukun Chen:** data curation, formal analysis, writing – original draft, writing – review and editing, investigation. **Xiaofang Gong:** data curation, formal analysis, investigation. **Xiaobin Xiong:** formal analysis, writing – review and editing. **Gangjin Liu:** methodology, investigation. **Zhiye Wang:** methodology, resources. **Ying Zhu:** funding acquisition, supervision, writing – review and editing.

## Funding

Financial support for this investigation was provided by the Key Scientific and Technological Achievements Transformation Project of Ningxia Hui Autonomous Region, China (Grant No. 2025CJE09073), the Major Special Program of Gansu Academy of Sciences, China (Grant No. 2023ZDZX‐01), the Key Talent Program of Gansu Province, China (Grant No. 2025RCXM056), the Science and Technology Program of Gansu Province, China (Grant No. 2025QYXQ‐07), the Science and Technology Program of Gansu Province, China (Grant No. 24JRRA1137), the Science and Technology Program of Shandong Province, China (Grant No. YDZX2024033), the Young Scientists Fund Project of Gansu Academy of Sciences (Grant No. 2024QN‐13).

## Conflicts of Interest

The authors declare no conflicts of interest.

## Supporting information


**Figure S1:** Rarefaction curves of OTUs for (a) bacteria and (b) fungal.

## Data Availability

The data that support the findings of this study are available from the corresponding author upon reasonable request.
