# Supplementary figures and images for: Microbial Community Restructuring Enhances Composting Efficiency: Synergistic Roles of Thermal Cycling and Fungal Inoculants ( Fomes lignosus and Penicillium glabrum ) in Metabolic Adaptation
Source: Microb Biotechnol. 2025 Dec 26;18(12):e70290. doi: 10.1111/1751-7915.70290 (PMC12742449; doi:10.1111/1751-7915.70290)

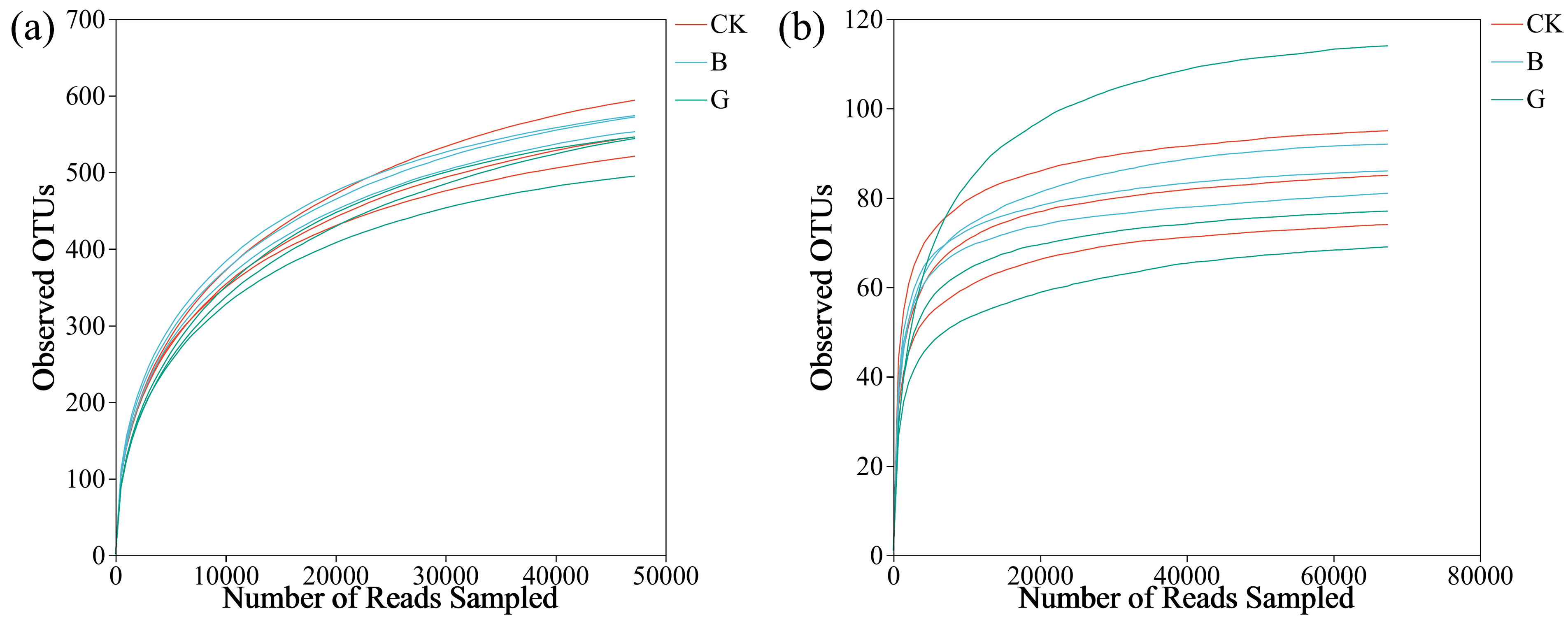

Supplement: Supplementary file 1 — Figure S1: Rarefaction curves of OTUs for (a) bacteria and (b) fungal. [file MBT2-18-e70290-s001.tif]
